# Effect of the Application Date of Fertilizer Containing Silicon and Potassium on the Yield and Technological Quality of Sugar Beet Roots

**DOI:** 10.3390/plants10020370

**Published:** 2021-02-15

**Authors:** Arkadiusz Artyszak, Dariusz Gozdowski, Alicja Siuda

**Affiliations:** Institute of Agriculture, Warsaw University of Life Sciences–SGGW, 159 Nowoursynowska St., 02-776 Warsaw, Poland; dariusz_gozdowski@sggw.edu.pl (D.G.); alicja1b@onet.eu (A.S.)

**Keywords:** sugar beet, drought, foliar application, potassium, silicon

## Abstract

Water shortage and drought are a growing problem in Europe. Therefore, effective methods for limiting its effects are necessary. At the same time, the “field to fork” strategy adopted by the European Commission aims to achieve a significant reduction in the use of plant protection products and fertilizers in the European Union. In an experiment conducted in 2018–2020, the effect of the method of foliar fertilization containing silicon and potassium on the yield and technological quality of sugar beet roots was assessed. The fertilizer was used in seven combinations, differing in the number and time of application. The best results were obtained by treating plants during drought stress. The better soil moisture for the plants, the smaller the pure sugar yield increase was observed. It is difficult to clearly state which combination of silicon and potassium foliar application is optimal, as their effects do not differ greatly.

## 1. Introduction

The “field to fork” strategy adopted by the European Commission places significant restrictions on the use of plant protection products and mineral fertilizers. This threatens reduction of the production; therefore, it is necessary to look for innovative technologies that could prevent it, and one of them may be silicon foliar application [1,2]. Application of silicon causes reduction of plant infestation by diseases [3,4,5,6,7,8,9,10] and pest [5,8,9,11,12,13]. It also increases the plant’s tolerance to drought [14,15,16,17,18,19,20,21,22,23,24,25], high and low temperature [14,25,26,27], shading [28], flooding with water [14], and deficiency of macro- and micro-nutrients [29,30,31]. Silicon can also reduce the harmful effects of soil salinity, which can be severe problem in some regions [4,32,33,34,35]. Moreover, silicon can reduce the negative impact of heavy metals on plants [36,37,38].

There is a relatively small number of studies on the foliar application of silicon in the cultivation of sugar beet [15,16,39,40,41,42]. The effects were better and stronger if abiotic and biotic stresses occurred in the plants. Nevertheless, an open question of agricultural producers regarding the technical details of foliar application requires an urgent answer: what is the optimal application date and what is the optimal number of treatments in sugar beet crop production?

Various forms of products containing silicon are available on the market. Unfortunately, products containing stabilized orthosilicic acid are usually much more expensive than potassium silicates. Therefore, potassium silicate was used in the experiment. The effect of the application of different doses and dates of foliar fertilization with Sarmin maKSi fertilizer containing potassium silicate (K_2_SiO_3_) for different periods of time on the root yield of sugar beet, the biological sugar yield, and the pure sugar yield was evaluated.

## 2. Results

The results were presented using means across three years (2018–2020). In each year, a different cultivar was sown, but of the same normal type of sugar beet. Moreover, results for each cultivar (year) were presented separately (Tables S1, S2, and S3). The plant density during harvest was 79.5–89.00 thousand per ha (Table 1). Treatments No. 1, 2, 3, i.e., treatments where the first foliar fertilization was applied in BBCH (Biologische Bundesanstalt, Bundessortenamt und CHemische Industrie growth stage scale) 16 stage, and 6 with foliar application of the tested product resulted in a significant increase in the root yield in relation to the control (without foliar fertilization). The effect of the fertilizer application date varied in years of the experiment. In 2018, significantly higher root yields than in treatment No. 0 were obtained in the treatments No. 5 and 6, where the first foliar fertilization was applied 7 days after BBCH 16 stage, in 2019, on all treatments except No. 4 and 7, and in 2020, only for treatment No. 6 (Table S1). 

The treatments where foliar applications were applied had no significant effect or significantly increased the sugar content in the roots as compared to the control treatment (Table 2). In the case of α-amino nitrogen, no significant effect was observed for most treatments, excluding treatments No. 2 and No. 6, where a significant reduction was observed, and in treatment No. 7, where a significant increase in content was observed. On the other hand, the content of potassium and sodium was significantly reduced as a result of the applied foliar nutrition, especially for potassium, which was the highest for the control. The value of the alkalinity coefficient was the highest for treatment No. 2 and the lowest for treatment No. 7.

The highest sugar content for cultivar Lavenda KWS (year 2018) was found in the treatment No. 3, for the cultivar Toleranza KWS (year 2019) in the treatment No. 1, and, for cultivar Jaromir (year 2020), all the tested treatments showed a similar value of this trait (Table S2). The differences in the sugar content in the roots should be explained rather by the course of weather conditions during the growing season, not by differences between cultivar, because they were very similar.

All treatments in which first foliar fertilization was applied no later than 7 days after BBCH 16 stage contributed to a significant increase in the biological sugar yield and the pure sugar yield as compared to treatment No. 0 (Table 3). Treatment No. 6 had significantly larger biological sugar yield and pure sugar yield in years 2018–2019 in comparison to treatment No. 0 (Table S3). In year 2019, treatments No. 1, 2, 3, i.e., treatments where the first foliar fertilization was applied in BBCH 16 stage, and 5 had significantly higher sugar yields. However, in 2020, no significant differences were found between the studied treatments.

The foliar application of silicon and potassium caused a significant increase in the fresh mass of the root, except for treatments No. 1 and No. 2 (Table 4). In 2018 and in 2020, plants in treatments No. 4, 6, and 7 had significantly higher fresh root mass than in treatment No. 0, and in 2019, in treatments No. 1, 2, and 3 (Table S3). 

The fresh mass of leaves of a single plant was the most variable during the research period (coefficient of variation—CV = 52.4%) and the period of the lowest productivity of sugar (CV = 2.70%) (Table 5).

Table 6 shows the results based on the general linear model (GLM) for repeated measures where they were traits measured in the subsequent measurements. On the basis of these effects, we can conclude that a strong multivariate effect was observed for the year, as well all interactions with the repeated measures, which means that the effect was different for different variables.

## 3. Discussion

The final product in sugar beet production is pure sugar yield. The largest increases in pure sugar yield were obtained in 2018, when plants were exposed to drought stress in May. Smaller increases were observed in 2019, and their lack in 2020, when the value of the soil moisture was high and there was no drought stress. This confirms the results of previous studies, which prove that the effect of silicon applied on leaves is particularly visible in plants when they are exposed to strong stress factors [15,16,25].

Pure sugar yield is determined by the biological yield of sugar, which depends on the yield of roots and sugar content, as well as the content of molasses-forming components (α-amino nitrogen, potassium, and sodium ions). Root yield has the greatest influence on pure sugar yield [43]. The resulting increase in root yields occurs as a result of the fertlizer containing potassium silicate, despite the opinion that silicates only reduce biotic stress and have no effect on reducing abiotic stress, plant and root growth, leaf size, yield, and production quality [1]. The beneficial effect of foliar application of products containing various forms of silicon on the yield of sugar beet was found in previous studies. It was: marine calcite [15,39,40,41], mixture of poly- and ortho-silicic acids with the addition of Fe [15,40], choline stabilized orthosilicic acid with the addition of Ca [15], and silicon oxide nanoparticles formulated in propylene glycol with natural esters [16,42]. The potato tuber yield also increased due to the foliar application of marine calcite [44] stabilized orthosilicic acid with the addition of microelements [25,45], oligomeric silicic acid, and boric acid [4]. In some studies, the foliar application of choline stabilized orthosilicic acid with the addition of Ca did not increase the yield of potato tubers in relation to the control treatment [46].

In our current research, the technological quality of the roots usually did not change significantly, or it improved under the influence of the applied applications with the tested product; less often, the content of any molasses-forming component was significantly higher. This is confirmed by previous studies in which various forms of silicon were used for foliar application [15,16,39,40,41,42]. In potato cultivation, foliar application of silicon-containing products had a positive effect on tuber quality [25,44,45,46]. 

The experimental results also confirm the profitability of foliar application of marine calcite, choline stabilized orthosilicic acid with the addition of Ca, and mixture of poly- and ortho-silicic acids with the addition of Fe [47,48].

## 4. Materials and Methods

**Figure 1:**
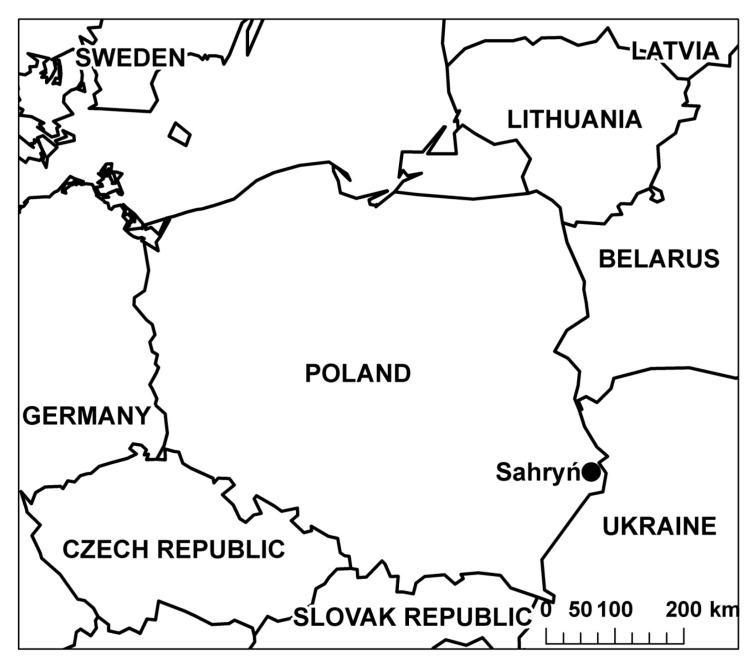
Location of the field experiment.

The experiment was carried out in Sahryń (50°41′ N, 23°46′ E) in 2018–2020 (Figure 1). The soil on which the experiment was located fulfill the very high soil requirements of sugar beet. The soil type was Calcic Chernozem (Aric, Siltic) (silty clay loam: clay—34%, sand—14%, silt—52%) [49]. Soil samples were collected at a depth of 0–30 cm immediately after harvesting of the forecrop. The soil chemical properties were evaluated at the District Chemical and Agricultural Stations in Warsaw-Wesoła. The following properties were evaluated: content of soil organic carbon (SOC) [50], pH potentiometrically in 1 M KCl [51], nitrate nitrogen (N-NO_3_) and ammonium nitrogen (NH_4_) [52], available macroelements (phosphorus [53], potassium [54], magnesium [55]), and microelements (boron [56], copper [57], iron [58], manganese [59], and zinc [60]). The same analyses were conducted after the harvest of sugar.

The soil samples collected for the 0–30 cm layer of depth after harvesting of the forecrop were analyzed. These samples contained 1.37–2.76% soil organic carbon (Table 7). The soil reaction was neutral or alkaline. Content of N-NO_3_ was in the range of 18.4–52.4 mg kg^−1^, N-NH_4_ content, was in the range of 1.58–3.33 mg kg^−1^, and mineral nitrogen content was in the range of 93–217 kg ha^−1^. The available contents of plants elements were as follows: P—44–99, K—62–133, Mg—60–99, B—2.2–5.6, Cu—6.9–8.8, Fe—490–735, Mn—157–192, and Zn—5.9–8.0 mg kg^−1^.

After the harvest of sugar beet, the soil contained 1.02–2.82% soil organic carbon, which was characterized by a pH in the range of 7.1–7.5. Content of N-NO_3_ was in the range of 22.7–84.3 mg kg^−1^, N-NH_4_ content was in the range of <1.00–1.54 mg kg^−1^, and mineral nitrogen was in the range of 89–335 kg ha^−1^. The available contents of phosphorus (P), potassium (K), and magnesium (Mg) were 12–66, 37–137, and 65–90 mg kg^−1^, respectively, while the contents of microelements were as follows: B—1.2–6.8, Cu—5.2–8.8, Fe—443–900, Mn—136–167, and Zn—4.6–9.0 mg kg^−1^. The foliar application of the tested potassium silicate did not affect the soil’s nutrient content because the amounts of potassium and silicon introduced were small, and most of them ended up on plant leaves during the spraying.

**Figure 2:**
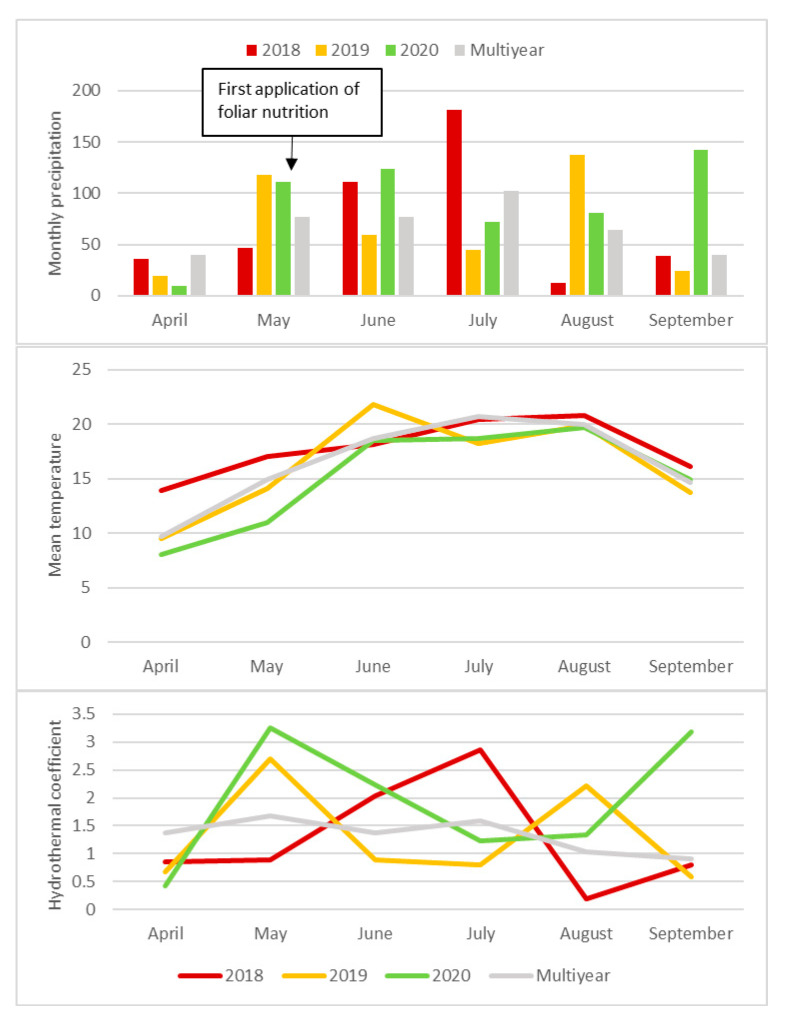
Weather conditions (monthly precipitations in mm, mean temperature in °C and hydrothermal coefficient k) during the growing season of sugar beet (2018–2020). Multiyear precipitation from year: 1991–2020 and temperature from 2002–2020. Source: own study based on data from Institute IMGW-PIB (Instytut Meteorologii i Gospodarki Wodnej – Państwowy Instytut Badawczy – Institute of Meteorology and Water Management – National Research Institute), Strzyżów Sugar Factory.

The amount of precipitation during the growing season (April−September) was from 426 mm in 2019 to 540 mm in 2020 (Figure 2). Sugar beet has the greatest water requirements in July and August. The largest rainfall deficit in July occurred in 2019, and in August, a year earlier. In May, before and during the foliar application of silicon and potassium, the plants were exposed to drought stress in 2018.

The characteristics of crop management in the experiment are presented in Table S4. In subsequent years, three different cultivars of sugar beet were sown, which are similar in terms of most of the traits, and the cultivars belong to the normal type of sugar beet.

Sowing was performed with a precision seed drill. The row spacing was 45 cm, the distances in the row were 15 cm, and the depth was 2–2.5 cm. Weed, disease, and pest control was conducted in accordance with the plant protection recommendations of the Institute of Plant Protection—National Research Institute in Poznań (Poland). 

In this study, foliar fertilizer Sarmin maKSi, which contains potassium silicate (Si—150 g dm^−3^, K—125 g dm^−3^), was applied. The experimental layout is presented in Table 8, including the total doses of Si and K by foliar application (per 1 ha).

In addition, a total of 600–630 g boron ha^–1^ was applied by foliar application in the whole experiment (2 × 300–315 g B ha^–1^).

The experiment in each year was based on a one factorial randomized block design, where different combination of dates and times of application were treated as one combined factor. However, due to the lack of significant block influence in the statistical analyses, the block effect was omitted, i.e., the analyses were performed for a completely randomized design.

In 2018, the foliar application was performed on May 22, May 29, and June 5; in 2019, on May 25, May 31, and June 8; and, in 2020, on May 23, May 30, and June 6. Spraying was performed with an Apollo trailed sprayer (Krukowiak). The dose of water in each spraying was 250 dm^3^ ha^–1^, and the concentration of the fertilizer was 2%. In the subsequent sprayings, we estimated that about 50% (1st spray), 75% (2nd spray), and 90–95% (3rd spray) of liquid was left on the plants. The effect of foliar fertilization in this case is much higher in comparison to uptake of the fertilizer by the roots.

The number of repetitions was 4, and the total number of plots was 32. Each plot included 6 rows. The dimensions of a single plot were a length of 16 m and width of 2.7 m (43.2 m^2^), of which 21.6 m^2^ was for harvesting (3 middle rows). During harvest, the plants were topped by hand on the three middle rows, and the leaves were weighed. The roots were then counted, dug up, and weighed. During the harvest, each plot was collected in accordance with the Polish Standard [61].

The root samples were pulped in the Plant Breeding Station of the Kutno Sugar Beet Breeding Company in Śmiłów. In Straszków (the Kutno Sugar Beet Breeding Company) on the automatic Venema technological line [62], the sugar content polarimetrically [63], the α-amino nitrogen by fluorometric methods [64], and the K and Na by photoelectric flame photometry [63].

The measurements performed in the experiments were as follows:

- Plant density at harvest (thousand plants ha^−1^);
- Root yield (t ha^−1^);
- Yield of leaves (t ha^−1^);
- Yield of fresh biomass (t ha^−1^) as a sum of the root yield (t ha^−1^) and yield of leaves (t ha^−1^);
- Harvest index (HI) as a ratio of root yield to fresh biomass;
- Foliage coefficient as a ratio of yield of leaves to root yield;
- Content of sucrose in the roots (%);
- Content of α-amino nitrogen in the roots (mmol kg^−1^);
- Content of potassium (K) in the roots (mmol kg^−1^);
- Content of sodium (Na) in the roots (mmol kg^−1^);
- Alkalinity coefficient = (content of K (mmol kg^−1^) + content of Na (mmol kg^−1^))/content of α-amino nitrogen (mmol kg^−1^) [65];
- Sugar yield losses (%) = standard molasses losses (%) + 0.6 (%);
- Standard molasses losses (%) = 0.012 × (K + Na) + 0.024 (α-amino nitrogen) + 0.48 [66], where the content of K, Na, and α-amino nitrogen are given in mmol kg^−1^ of pulp;
- Refined sugar content (%) = sucrose content (%) − sugar yield losses (%);
- Sugar productivity (%) = refined sugar content (%)/sugar content (%) × 100;
- Biological sugar yield (t ha^−1^) = root yield (t ha^−1^) × content of sugar in roots (%);
- Pure sugar yield (t ha^−1^) = root yield (t ha^−1^) × [content of sugar (%) − sugar yield losses (%)] [66];
- Fresh biomass of root (kg) as a ratio of root yield (kg) and the number of plants per plot at harvest;
- Fresh biomass of leaves per plant (kg) as a ratio of (kg) and the number of plants per plot at harvest;
- Fresh weight of individual plant as the sum of fresh root mass (kg) and leaves of a single plant (kg).

The data were subjected to ANOVA (analysis of variance) and Tukey’s HSD (honestly significant difference test) procedure of multiple comparison of means. The analyses were performed using Statistica 13 software (TIBCO Software Inc., Palo Alto, CA, USA) at a significance level of 0.05. Basic statistical parameters, such as range (min−max), standard deviation (SD), and coefficient of variation (CV), were calculated for all variables.

Moreover, a general linear model (GLM) for repeated measures was applied, where the repeated measures were traits measured in the subsequent measurements. For such analysis, the following variables were selected: plant density, root yield, yield of leaves, technological yield of sugar, sugar content in roots, and sugar efficiency.

## 5. Conclusions

The foliar application of potassium silicate in sugar beet cultivation gives the best production results when plants are exposed to drought stress. The better soil moisture for the plants, the increase of the pure sugar yield is lower. It is difficult to indicate which of the treatment is the most optimal. The aim of further research should be an explanation of the mechanism of action of potassium silicate on plants.

## Figures and Tables

**Table 1:** Means and standard deviations (SD) for yield and yield-related traits of sugar beet (from 2018 to 2020).

Fertilization Treatment	Plant Density at Harvest, Thousand pcs. ha^−1^		Root Yield, t ha^−1^		Yield of Leaves, t ha^−1^		Total Yield (Roots + Leaves), t ha^−1^		Harvest Index		Foliage Index	
	mean	SD	mean	SD	mean	SD	mean	SD	mean	SD	mean	SD
0	88.66 a ^1^	14.49	76.57 b	5.96	41.98 a	17.08	118.55 c	22.24	0.66 d	0.09	0.54 a	0.19
1	89.00 a	13.37	84.93 a	7.39	43.41 a	15.04	128.34 abc	18.28	0.67 bcd	0.07	0.51 ab	0.18
2	86.11 ab	10.54	85.00 a	9.86	39.51 ab	16.23	124.51 bc	24.55	0.70 ab	0.08	0.46 bc	0.16
3	85.42 ab	15.00	84.37 a	13.69	44.33 a	20.17	128.70 ab	27.96	0.67 cd	0.09	0.53 a	0.23
4	84.72 ab	18.74	87.55 ab	7.31	41.18 ab	13.46	128.73 ab	18.53	0.69 abc	0.07	0.47 bc	0.14
5	87.15 a	14.59	87.05 ab	11.99	41.84 a	20.76	128.89 ab	31.88	0.69 ab	0.08	0.46 bc	0.18
6	82.52 ab	17.51	92.20 a	11.05	43.20 a	19.14	135.40 a	29.29	0.70 ab	0.08	0.45 bc	0.16
7	79.51 b	17.68	83.83 b	13.74	35.98 b	12.73	119.81 bc	24.49	0.71 a	0.06	0.42 c	0.12

^1^ Within each column, means associated to different letters are significantly different at *p* < 0.05 according to Tukey’s test.

**Table 2:** Means and standard deviations (SD) of technological quality of sugar beet roots (from 2018 to 2020).

Fertilization Treatment	Sugar Content, %		mmol kg^−1^						Alkalinity Factor		Standard Molasses Losses, %		Sugar Yield Losses, %		Refined Sugar Content, %		Sugar Productivity, %	
			α-Amino Nitrogen		K		Na											
	mean	SD	mean	SD	mean	SD	mean	SD	mean	SD	mean	SD	mean	SD	mean	SD	mean	SD
0	17.01 bc ^1^	1.20	28.89 b	4.73	39.06d a	2.55	3.73 a	0.87	1.51 ab	0.21	1.69 ab	0.13	2.29 ab	0.13	14.72 cd	1.31	86.45 de	1.64
1	17.46 a	1.64	28.68 bc	10.44	36.53 cc	7.32	2.84 c	0.74	1.48 bc	0.34	1.64 bc	0.34	2.24 bc	0.34	15.21 a	1.97	86.90 abcd	3.10
2	17.39 a	1.16	26.83 cd	6.92	37.82 cda	4.43	3.40 ab	0.58	1.62 a	0.41	1.62 c	0.20	2.22 c	0.20	15.18 a	1.36	87.13 ab	1.97
3	17.24 ab	1.16	27.96 bcd	8.64	36.46 cc	4.03	3.40 ab	0.68	1.55 ab	0.44	1.63 c	0.24	2.23 c	0.24	15.01 abc	1.36	86.95 abc	2.13
4	17.02 bc	1.45	28.78 b	7.72	37.48 ccda	4.29	3.01 bc	0.53	1.48 bc	0.32	1.66 abc	0.23	2.26 abc	0.23	14.76 bcd	1.67	86.55 cde	2.47
5	17.05 bc	1.47	29.17 bc	7.04	35.50 ac	3.62	2.94 bc	0.52	1.38 c	0.29	1.64 bbc	0.20	2.24 bc	0.20	14.81 bcd	1.67	86.68 bcd	2.30
6	17.23 ab	1.53	26.44 d	8.09	33.83 a	5.57	2.96 bc	0.47	1.46 bc	0.29	1.56 d	0.26	2.16 d	0.26	15.08 ab	1.76	87.29 a	2.55
7	16.86 c	1.63	30.83 a	8.15	36.87 cc	6.60	3.74 a	0.97	1.37 c	0.25	1.71 a	0.27	2.31 d	0.27	14.56 d	1.86	86.09 e	2.75

^1^ Within each column, means associated to different letters are significantly different at *p* < 0.05 according to Tukey’s test.

**Table 3:** Means and standard deviations (SD) of sugar yield of sugar beet (from 2018 to 2020).

Fertilization Treatment	Biological Sugar Yield, t ha^−1^		Pure Sugar Yield, t ha^−1^	
	mean	SD	mean	SD
0	13.05 c ^1^	1.58	11.30 c	1.54
1	14.89 ab	2.35	13.00 ab	2.46
2	14.84 ab	2.36	12.96 ab	2.31
3	14.63 b	3.00	12.76 b	2.85
4	14.92 ab	1.84	12.94 ab	1.87
5	14.88 ab	2.59	12.93 ab	2.47
6	15.91 a	2.40	13.93 a	2.39
7	14.18 bc	2.84	12.25 bc	2.71

^1^ Within each column, means associated with different letters are significantly different at *p* < 0.05 according to Tukey’s test.

**Table 4:** Morphological features of sugar beet (means from 2018 to 2020).

Fertilization Treatment	Fresh Weight of Individual Root, kg	Fresh Weight of Leaves per Plant, kg	Fresh Weight of Individual Plant, kg
0	0.89 d ^1^	0.51 cd	1.40 c
1	0.98 cd	0.51 cd	1.49 bc
2	1.00 bcd	0.47 d	1.47 bc
3	1.03 bc	0.58 b	1.61 ab
4	1.10 abc	0.53 bcd	1.63 ab
5	1.02 bc	0.50 cd	1.52 bc
6	1.18 a	0.56 bc	1.74 a
7	1.11 ab	0.48 d	1.59 ab

^1^ Within each column, means associated with different letters are significantly different at *p* < 0.05 according to Tukey’s test.

**Table 5:** Basic statistics for the studied traits of sugar beet in years 2018–2020 for all treatments together.

Element	Mean	Minimum	Maximum	SD	Coefficient of Variation, %
Plant density at harvest, thousand pcs. ha^−1^	85.39	55.56	113.89	15.16	17.76
Root yield, t ha^−1^	85.19	61.89	110.28	10.92	12.82
Yield of leaves, t ha^−1^	41.43	14.92	77.22	16.61	40.09
Total yield (roots + leaves), t ha^−1^	126.62	78.67	180.83	24.68	19.49
Harvest index	0.68	0.52	0.82	0.08	11.34
Foliage index	0.48	0.21	0.92	0.17	35.71
Sugar content in the roots, %	17.16	14.72	19.88	1.38	8.03
The content of α-amino nitrogen in the roots, mmol kg^−1^	28.45	14.80	42.40	7.68	27.00
Potassium content in the roots, mmol kg^−1^	36.69	27.50	48.80	5.06	13.78
Sodium content in the roots, mmol kg^−1^	3.25	1.90	5.60	0.75	22.97
Alkalinity factor	1.48	0.99	2.39	0.33	21.97
Standard molasses losses, %	1.64	1.20	2.11	0.24	14.34
Sugar yield losses, %	2.24	1.80	2.71	0.24	10.50
Refined sugar content, %	14.92	12.17	18.02	1.59	10.67
Productivity of sugar, %	86.75	81.91	90.65	2.34	2.70
Biological sugar yield, t ha^−1^	14.66	9.99	19.46	2.44	16.66
Pure sugar yield, t ha^−1^	12.76	8.32	17.39	2.38	18.64
Fresh weight of individual root, kg	1.04	0.67	1.82	0.28	27.37
Fresh weight of leaves per plant, kg	0.52	0.19	1.26	0.27	52.41
Fresh weight of individual plant, kg	1.56	0.92	2.96	0.53	33.82

**Table 6:** Effects based on general linear model which include effect of repeated measures.

	SS	df	MS	F	*p*
Intercept	1,704,958	1	1,704,958	32,565.06	<0.001
Year	7809	2	3904	74.57	<0.001
Treatment	737	7	105	2.01	0.065
Year*treatment	1028	14	73	1.40	0.175
MSE1	3770	72	52		
Repeated measures ^1^	615,935	5	123,187	4837.31	<0.001
Repeated measures*year	31,705	10	3170	124.50	<0.001
Repeated measures*treatment	2388	35	68	2.68	<0.001
Repeated measures*year*treatment	4078	70	58	2.29	<0.001
MSE2	9168	360	25		

^1^ Repeated measures are selected variables measured in subsequent measurements, such as: plant density, root yield, yield of leaves, technological yield of sugar, sugar content in roots, and sugar efficiency.

**Table 7:** Soil properties before establishing the experiment with sugar beet and after the harvesting (2017/2018–2019/2020).

Year	SOC, %	pH_KCl_	mg kg^−1^		Nmin, kg ha^−1^	mg kg^−1^							
			N-NO_3_	N-NH_4_		P	K	Mg	B	Cu	Fe	Mn	Zn
before													
2017	1.66	7.5	36.2	1.58	147	87	62	69	2.2	7.3	490	167	5.9
2018	2.76	7.3	18.4	3.11	93	91	133	99	5.6	8.8	630	157	8.0
2019	1.37	7.2	52.4	3.33	217	44	116	60	2.3	6.9	735	192	6.4
after													
2018	2.40	7.5	84.3	1.54	335	12	37	65	5.8	8.8	900	159	9.0
2019	2.82	7.4	47.4	<1.00	185	66	71	90	6.8	8.3	606	167	6.0
2020	1.02	7.1	22.7	<1.00	89	38	137	73	1.2	5.2	443	136	4.6

**Table 8:** Description of the studied fertilization treatments.

Fertilization Treatment	Spraying Date			
	6 Leaf Stage (BBCH 16)	7 Days Later	14 Days Later	Total Dose of Si and K Applied by Foliar Application per Season per 1 ha **
Control (0)	–	–	–	Without foliar Si and K fertilization
1	0.5 dm^3^ ha^−1^ *	–	–	75 g Si, 62.5 g K
2	0.5 dm^3^ ha^−1^	0.5 dm^3^ ha^−1^	–	150 g Si, 125 g K
3	0.5 dm^3^ ha^−1^	0.5 dm^3^ ha^−1^	0.5 dm^3^ ha^−1^	225 g Si, 187.5 g K
4	0.5 dm^3^ ha^−1^	–	0.5 dm^3^ ha^−1^	150 g Si, 125 g K
5	–	0.5 dm^3^ ha^−1^	–	75 g Si, 62.5 g K
6	–	0.5 dm^3^ ha^−1^	0.5 dm^3^ ha^−1^	150 g Si, 125 g K
7	–	–	0.5 dm^3^ ha^−1^	75 g Si, 62.5 g K

* In each dose of 0.5 dm^3^ Sarmin maKSi, 75 g Si and 62.5 g K were added. ** Total dose of Si and K per season applied by foliar fertilization.

## Data Availability

The data presented in this study are available on request from the corresponding author. The data are not publicly available due to ongoing unpublished research.

## Supplementary Materials

The following are available online at https://www.mdpi.com/2223-7747/10/2/370/s1, Table S1. Means and standard deviations (SD) for yield and yield-related traits of sugar beet separately for each cultivar (year). Table S2. Means and standard deviations (SD) of technological quality of sugar beet roots separately for each cultivar (year). Table S3. Means and standard deviations (SD) of sugar yield of sugar beet and morphological features of sugar beet separately for each cultivar (year). Table S4. Crop management in the field experiments (2017/2018–2019/2020).
