# Supplementary material for: Effect of the Application Date of Fertilizer Containing Silicon and Potassium on the Yield and Technological Quality of Sugar Beet Roots
Source: Plants (Basel). 2021 Feb 15;10(2):370. doi: 10.3390/plants10020370 (PMC7918965; doi:10.3390/plants10020370)
Supplement: Supplementary file 1 [file plants-10-00370-s001.pdf]

Table S1. Means and standard deviations (SD) for yield and yield-related traits of sugar beet separately for each cultivar (year).

Fertilization treatment	Plant density at harvest, thousand pcs. ha^{-1}		Root yield, t ha^{-1}		Yield of leaves, t ha^{-1}		Total yield (roots + leaves), t ha^{-1}		Harvest index		Foliage index	
	mean	SD	mean	SD	mean	SD	mean	SD	mean	SD	mean	SD
					Lavenda KWS - year 2018							
0	70.83ab[1]	4.81	81.39c	3.37	60.97ab	3.21	142.36bc	6.58	0.57cd	0.01	0.75ab	0.01
1	72.22ab	6.42	84.17bc	3.21	56.04bc	13.55	140.21c	10.34	0.60bc	0.07	0.67bc	0.19
2	76.39a	4.81	89.51abc	9.38	54.03bc	6.09	143.54bc	15.48	0.62ab	0.00	0.60cd	0.01
3	66.67ab	6.42	86.39bc	19.73	70.49a	7.78	156.88abc	27.50	0.55d	0.03	0.83a	0.10
4	63.89b	9.62	95.21abc	0.72	53.89bc	1.60	149.10abc	2.33	0.64ab	0.01	0.57cd	0.01
5	76.39a	8.02	99.17ab	2.57	65.97ab	11.39	165.14ab	13.95	0.60bc	0.04	0.66bc	0.10
6	66.67ab	12.83	104.58a	4.33	63.26ab	10.66	167.85a	15.00	0.63ab	0.03	0.60cd	0.08
7	61.11b	6.42	94.38abc	18.36	47.36c	6.90	141.74bc	25.26	0.66a	0.01	0.51d	0.03
					Toleranza KWS - year 2019							
0	99.31a	5.73	77.40b	1.16	43.16ab	3.16	120.56c	2.15	0.64b	0.02	0.56a	0.05
1	97.22a	6.00	90.04a	1.02	47.36a	5.14	137.40a	4.53	0.66b	0.03	0.53a	0.06
2	94.44a	11.34	88.80a	9.42	45.59a	5.57	134.39ab	14.69	0.66b	0.01	0.51a	0.02
3	92.01a	3.65	91.15a	8.25	35.61b	2.80	126.75abc	9.66	0.72a	0.02	0.39b	0.04
4	104.17a	6.61	85.42ab	4.60	43.18ab	11.96	128.59abc	9.85	0.67ab	0.08	0.51a	0.15
5	102.08a	14.76	86.91a	10.47	39.44ab	4.81	126.35abc	13.61	0.69ab	0.03	0.46ab	0.06
6	101.39a	1.60	89.02a	1.88	45.51a	1.35	134.52ab	2.13	0.66b	0.01	0.51a	0.02
7	97.22a	9.89	82.74ab	6.75	39.57ab	7.20	122.31bc	6.62	0.68ab	0.05	0.48ab	0.11
					Jaromir - year 2020							
0	95.83a	8.41	70.93b	6.59	21.81ab	4.89	92.74b	10.81	0.77abcd	0.03	0.31abcd	0.05
1	97.57a	3.82	80.59ab	11.32	26.82a	4.34	107.41a	14.71	0.75cd	0.02	0.33ab	0.04
2	87.50ab	5.89	76.67ab	6.41	18.92b	1.44	95.59ab	5.09	0.80a	0.03	0.25d	0.04
3	97.57a	6.84	75.56ab	7.75	26.90a	2.03	102.46ab	5.96	0.74d	0.03	0.36a	0.06
4	86.11ab	8.10	82.02ab	7.00	26.47a	3.16	108.49a	7.53	0.76bcd	0.03	0.32abc	0.05
5	82.99b	4.86	75.07ab	4.91	20.11b	3.72	95.18ab	8.47	0.79ab	0.02	0.27cd	0.04
6	79.51b	11.64	83.01a	9.76	20.84b	4.18	103.85ab	13.62	0.80a	0.02	0.25d	0.03
7	80.21b	11.70	74.37ab	6.37	21.03b	2.69	95.40ab	8.57	0.78abc	0.02	0.28bcd	0.03

[1]Within each column, means associated with different letters are significantly different at $p < 0.05$ according to Tukey's test.

Table S2. Means and standard deviations (SD) of technological quality of sugar beet roots separately for each cultivar (year).

Fertili-zation treatment	Sugar content, %		α-amino nitrogen mmol kg^{-1}		K mmol kg^{-1}		Na mmol kg^{-1}		Alkali-nity factor		Standard molasses losses, %		Sugar yield losses, %		Refined sugar content, %		Sugar productivity, %	
	mean	SD	mean	SD	mean	SD	mean	SD	mean	SD	mean	SD	mean	SD	mean	SD	mean	SD
								Lavenda KWS - year 2018										
0	16.93abc[1]	0.37	30.55a	2.02	38.95a	2.02	4.50a	0.58	1.42a	0.05	1.73a	0.07	2.33a	0.07	14.60ab	0.44	86.20cd	0.69
1	17.20a	0.43	29.90a	1.39	36.35bc	0.52	3.50bc	0.81	1.34ab	0.07	1.68ab	0.03	2.28ab	0.03	14.92a	0.40	86.76bc	0.16
2	17.14ab	0.07	28.45ab	2.60	35.65bc	2.14	3.95ab	0.64	1.40a	0.07	1.64ab	0.08	2.24ab	0.08	14.90a	0.15	86.94ab	0.52
3	17.27a	0.18	30.85a	5.83	36.75abc	0.40	3.50bc	0.01	1.34ab	0.24	1.70ab	0.14	2.30ab	0.14	14.97a	0.33	86.65bc	0.98
4	16.74bc	0.06	27.90ab	0.81	37.25ab	0.06	3.40bc	0.46	1.46a	0.06	1.64b	0.01	2.24b	0.01	14.50ab	0.08	86.63bc	0.13
5	16.54c	0.24	31.05a	0.06	34.40cd	1.50	3.30bc	0.01	1.21bc	0.05	1.68ab	0.02	2.28ab	0.02	14.26bc	0.26	86.23bcd	0.32
6	16.88abc	0.17	25.00b	0.46	31.70e	2.31	2.95c	0.17	1.38a	0.07	1.50c	0.04	2.10c	0.04	14.78a	0.13	87.59a	0.11
7	16.07d	0.57	31.55a	2.14	32.70de	2.42	3.70b	0.12	1.16c	0.15	1.67ab	0.02	2.27ab	0.02	13.80c	0.54	85.84d	0.35
								Toleranza KWS - year 2019										
0	18.39c	0.13	23.45a	0.06	36.95a	1.33	3.60a	0.01	1.73b	0.06	1.53a	0.01	2.13a	0.01	16.26d	0.11	88.42c	0.00
1	19.45a	0.50	15.95e	1.33	28.30d	0.58	2.15c	0.29	1.92ab	0.14	1.23e	0.04	1.83e	0.04	17.62a	0.46	90.60a	0.06
2	18.85bc	0.03	18.40cd	0.12	35.90ab	5.54	3.25ab	0.29	2.13a	0.30	1.39bc	0.07	1.99bc	0.07	16.86bc	0.04	89.44b	0.37
3	18.51c	0.53	17.60de	0.46	33.30ab	0.81	3.60a	1.15	2.10a	0.04	1.35cd	0.02	1.95cd	0.02	16.56cd	0.54	89.48b	0.38
4	18.79bc	0.23	20.35bc	2.48	33.50ab	1.96	2.50bc	0.01	1.80b	0.32	1.40bc	0.04	2.00bc	0.04	16.79bcd	0.27	89.35b	0.32
5	18.91abc	0.27	20.30bc	0.81	32.95b	2.14	2.75bc	0.40	1.76b	0.02	1.40bc	0.04	2.00bc	0.04	16.91bc	0.23	89.45b	0.06
6	19.11ab	0.66	17.80de	0.46	29.15cd	1.91	2.95ab	0.75	1.81b	0.20	1.29de	0.02	1.89de	0.02	17.21ab	0.64	90.09a	0.24
7	18.97abc	0.40	21.50ab	2.77	32.50bc	2.66	2.90abc	0.23	1.66b	0.10	1.42b	0.10	2.02b	0.10	16.95bc	0.50	89.34b	0.73
								Jaromir - year 2020										
0	15.70a	0.50	32.68c	3.94	41.28ab	2.34	3.08b	1.02	1.38a	0.26	1.80c	0.07	2.40c	0.07	13.30ab	0.46	84.73ab	0.41
1	15.72a	0.22	40.18a	1.38	44.95a	3.32	2.88b	0.22	1.19ab	0.08	2.02a	0.06	2.62a	0.06	13.10ab	0.23	83.34cd	0.42
2	16.19a	0.36	33.65c	2.96	41.90ab	1.82	3.00b	0.29	1.34ab	0.11	1.83bc	0.08	2.43bc	0.08	13.77a	0.43	85.00a	0.81
3	15.95a	0.54	35.43bc	3.30	39.33b	5.87	3.10b	0.44	1.20ab	0.15	1.84bc	0.13	2.44bc	0.13	13.51ab	0.43	84.71ab	0.42
4	15.53a	0.60	38.10ab	0.29	41.68ab	4.37	3.13b	0.50	1.18ab	0.13	1.93ab	0.06	2.53ab	0.06	13.00b	0.63	83.67bcd	0.88
5	15.70a	0.65	36.15abc	2.53	39.15b	3.64	2.78b	0.76	1.16b	0.10	1.85bc	0.10	2.45bc	0.10	13.25ab	0.75	84.35abc	1.29
6	15.72a	0.48	36.53abc	1.33	40.63ab	2.79	2.98b	0.46	1.19ab	0.08	1.88bc	0.06	2.48bc	0.06	13.24ab	0.51	84.20abcd	0.76
7	15.55a	0.40	39.45ab	3.96	45.40a	1.12	4.63a	1.18	1.28ab	0.16	2.03a	0.09	2.63a	0.09	12.93b	0.47	83.09d	0.96

[1]Within each column, means associated with different letters are significantly different at $p < 0.05$ according to Tukey's test.

Table S3. Means and standard deviations (SD) of sugar yield of sugar beet and morphological features of sugar beet separately for each cultivar (year).

	**Biological sugar yield, t ha^{-1}**		**Pure sugar yield, t ha^{-1}**		**Fresh weight of roots per plant, kg**		**Fresh weight of leaves per plant, kg**		**Fresh weight of individual plant, kg**	
	**mean**	**SD**	**mean**	**SD**	**mean**	**SD**	**mean**	**SD**	**mean**	**SD**
					Lavenda KWS - year 2018					
0	13.79 b[1]	0.87	11.89 b	0.85	1.15 b	0.03	0.86 bc	0.01	2.01 bcd	0.04
1	14.48 b	0.92	12.57 b	0.82	1.18 b	0.15	0.77 c	0.12	1.94 cd	0.03
2	15.35 ab	1.67	13.35 ab	1.53	1.18 b	0.20	0.71 c	0.12	1.89 d	0.32
3	14.95 ab	3.57	12.98 ab	3.24	1.33 ab	0.42	1.07 a	0.22	2.40 ab	0.64
4	15.93 ab	0.18	13.80 ab	0.18	1.52 a	0.24	0.86 bc	0.15	2.38 abc	0.39
5	16.41 ab	0.66	14.15 ab	0.63	1.31 ab	0.10	0.86 bc	0.06	2.17 abcd	0.04
6	17.66 a	0.91	15.47 a	0.78	1.60 a	0.24	0.95 ab	0.02	2.56 a	0.27
7	15.24 ab	3.48	13.09 ab	3.05	1.53 a	0.14	0.77 c	0.03	2.31 abcd	0.17
					Toleranza KWS - year 2019					
0	14.23 b	0.28	12.59 c	0.25	0.78 d	0.05	0.43 ab	0.02	1.22 c	0.05
1	17.51 a	0.35	15.86 a	0.33	0.93 abc	0.05	0.49 a	0.08	1.42 ab	0.13
2	16.74 a	1.76	14.97 ab	1.60	0.95 ab	0.10	0.49 ab	0.07	1.43 a	0.17
3	16.86 a	1.55	15.09 ab	1.40	0.99 a	0.12	0.39 b	0.03	1.38 abc	0.14
4	16.05 ab	0.82	14.34 abc	0.73	0.82 cd	0.03	0.42 ab	0.13	1.24 bc	0.13
5	16.45 a	2.12	14.71 ab	1.89	0.86 bcd	0.13	0.39 b	0.06	1.25 abc	0.17
6	17.01 a	0.89	15.33 ab	0.84	0.88 abcd	0.03	0.45 ab	0.02	1.33 abc	0.04
7	15.71 ab	1.57	14.04 bc	1.51	0.86 bcd	0.09	0.41 ab	0.07	1.26 abc	0.10
					Jaromir - year 2020					
0	11.12 a	0.87	9.42 a	0.70	0.74 c	0.02	0.23 b	0.03	0.97 c	0.03
1	12.68 a	1.83	10.56 a	1.52	0.82 bc	0.09	0.27 ab	0.04	1.10 abc	0.12
2	12.43 a	1.27	10.57 a	1.18	0.88 abc	0.03	0.22 b	0.03	1.09 abc	0.03
3	12.07 a	1.51	10.22 a	1.23	0.78 bc	0.11	0.28 ab	0.03	1.05 cd	0.11
4	12.77 a	1.55	10.69 a	1.39	0.96 ab	0.11	0.31 a	0.02	1.26 ab	0.10
5	11.77 a	0.62	9.93 a	0.54	0.91 abc	0.07	0.24 ab	0.04	1.15 abc	0.10
6	13.06 a	1.75	11.01 a	1.56	1.06 a	0.22	0.27 ab	0.07	1.33 a	0.29
7	11.57 a	1.10	9.61 a	0.93	0.95 ab	0.22	0.27 ab	0.07	1.22 ab	0.29

[1]Within each column, means associated with different letters are significantly different at $p < 0.05$ according to Tukey's test.

Table S4. Crop management in the field experiments (2017/2018–2019/2020).

Date of harvest of forecrop	Yield of straw of forecrop, dt ha^{-1}	Date of tillage of stubble with a stubble aggregate	Soil fertilization	Date of pre-sowing cultivation with a cultivator	Cultivar of sugar beet	Sowing date	Date and dose of foliar fertilization	Harvest date
			**2017/2018**					
23.07.2017	69.0	27.07.2017<br>16.08.2017<br>14.09.2017<br>16.10.2017	16.10.2017 – Polifoska 6 (400 kg ha^{-1})<br>16.10.2017 – Potassium chloride (300 kg ha^{-1})<br>09.04.2018 – Salmag with boron (550 kg ha^{-1})<br>Total dose (kg ha^{-1}): N – 173, P – 34.8, K – 249, S - 11.2, Ca - 11.6, Mg – 13.2 and B – 1.1	09.04.2018	Lavenda KWS	11.04.2018	25.05.2018 – Adob Bor (2 dm^{3} ha^{-1})<br>08.06.2018 – Adob Bor (2 dm^{3} ha^{-1})	06.09.2018
			**2018/2019**					
09.07.2018	31.0	10.07.2018<br>10.08.2018<br>11.09.2018<br>15.10.2018	15.10.2018 – Polifoska 6 (400 kg ha^{-1})<br>15.10.2018 – Potassium chloride (300 kg ha^{-1})<br>9.03.2019 – Saletrzak Standard 27 with boron (500 kg ha^{-1})<br>Total dose (kg ha^{-1}): N – 159, P – 34.8, K – 249, S – 11.2, Ca – 7, Mg – 12 and B – 1	09.03.2019<br>29.03.2019	Toleranza KWS	30.03.2019	29.05.2019 – Boron Forte – 1.5 kg ha^{-1}<br>10.06.2019 – Boron Forte – 1.5 kg ha^{-1}	26.09.2019
			**2019/2020**					
13.07.2019	84.0	18.07.2019<br>19.08.2019<br>18.09.2019<br>26.10.2019	26.10.2019 – Polifoska 6 fertilizer (400 kg ha^{-1})<br>26.10.2019 – Potassium chloride (300 kg ha^{-1})<br>16.03.2020 – Saletrzak 27 standard with boron (500 kg ha^{-1})<br>Total dose (kg ha^{-1}): N – 159, P – 34.8, K – 249, S – 11.2, Ca – 7, Mg – 12 and B – 1.	16.03.2020<br>03.04.2020	Jaromir	04.04.2020	04.06.2020 – Agri-Green Bor (2 dm^{3} ha^{-1}) + Plonvit Burak (2 dm^{3} ha^{-1})<br>15.06.2020 – Agri-Green Bor (2 dm^{3} ha^{-1}) + Plonvit Burak (2 dm^{3} ha^{-1})	25.09.2020

Polifoska 6 fertilizer (6%N in ammonium form, 8.7% P as mono and diammonium phosphate, 24.9% K as potassium chloride, and 2.8% S as sulphate), Potassium chloride (49.8% K as potassium chloride); Salmag with boron (13.5% N in the ammonium form and 13.5% N in the nitrate form, 2.1% Ca, 2.4% Mg, 0.2% B); Saletrzak Standard 27 with boron (13.5% N in the ammonium form and 13.5% N in the nitrate form, 1.4% Ca, 2.4% Mg, 0.2% B);
Adob Bor (N – 78 g dm^{-3}, B – 150 g dm^{-3}); Agri-Green Bor (B – 150 g dm^{-3}) Boron Forte (3% N, 14.1% K, 21% B); Plonvit Burak (N – 190 g dm^{-3}, Mg – 15.1 g dm^{-3}, Na – 28.8 g dm^{-3}, S – 9.2 g dm^{-3}, B – 6.3 g dm^{-3}, Cu – 2.5 g dm^{-3}, Fe – 2.5 g dm^{-3}, Mn – 8.2 g dm^{-3}, Mo – 0.06 g dm^{-3}, Zn – 6.3 g dm^{-3}, Ti – 0.26 g dm^{-3})
